# Supplementary figures and images for: Longitudinal, Multi-Platform Metagenomics Yields a High-Quality Genomic Catalog and Guides an In Vitro Model for Cheese Communities
Source: mSystems. 2023 Jan 9;8(1):e00701-22. doi: 10.1128/msystems.00701-22 (PMC9948695; doi:10.1128/msystems.00701-22)

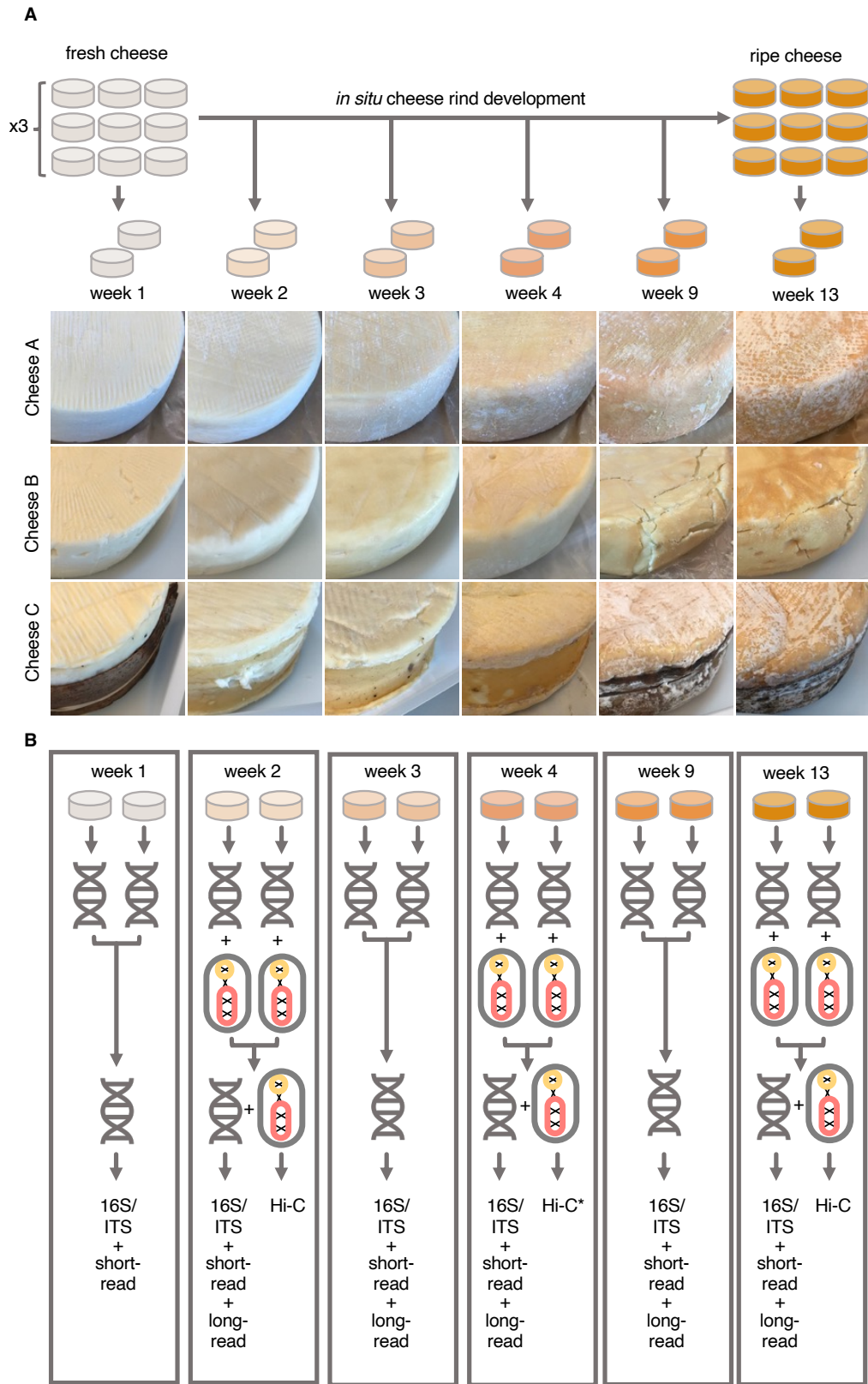

Supplement: FIG S1 [file msystems.00701-22-s0001.pdf]

**A**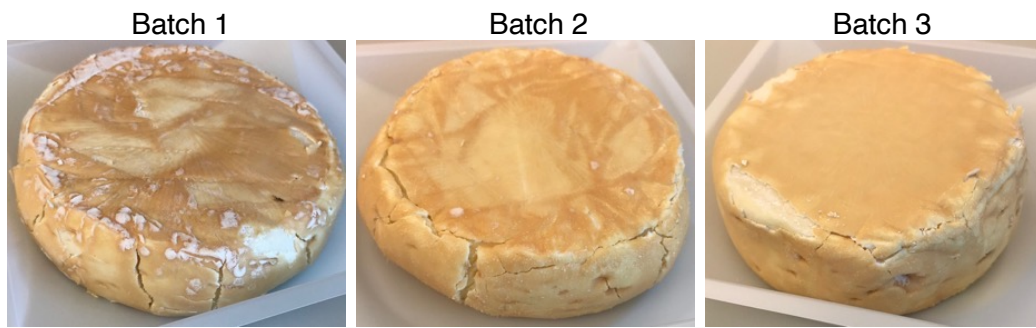**B**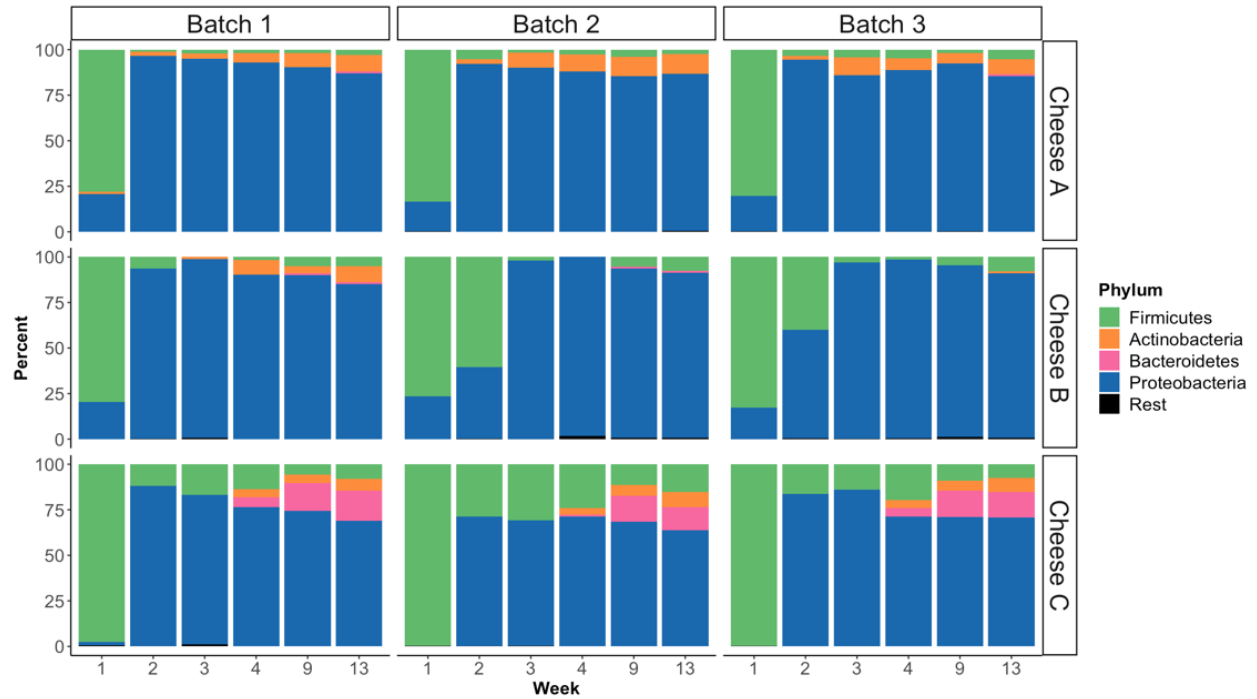**C**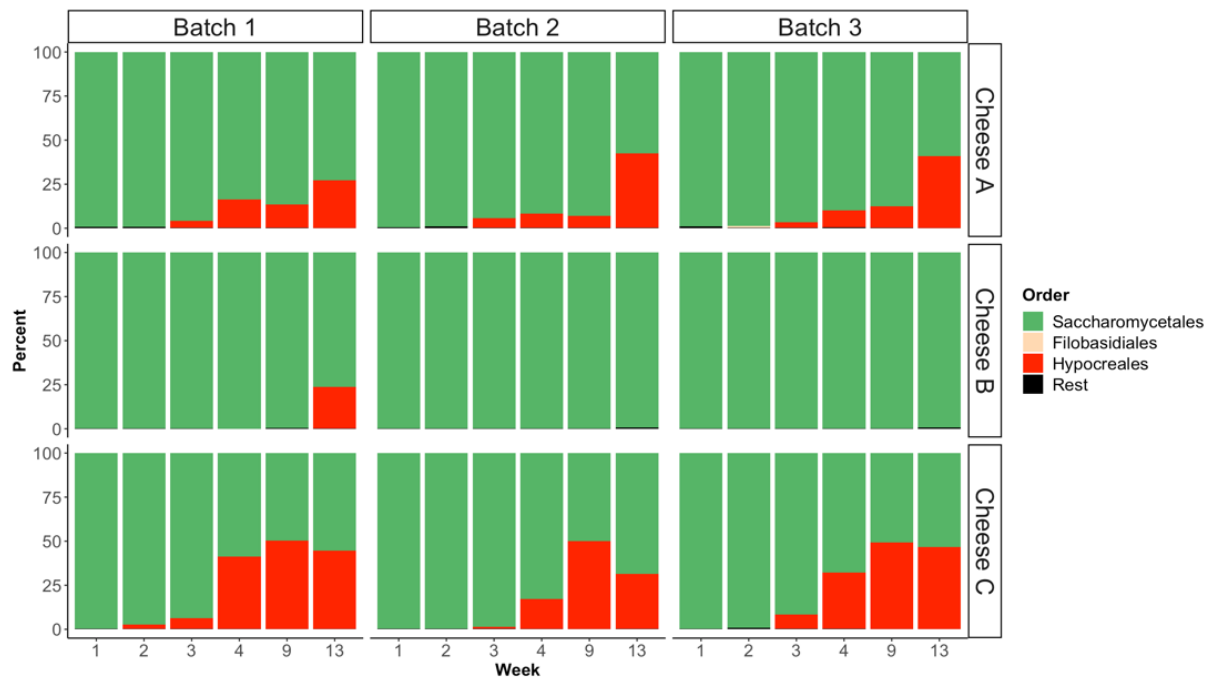

Supplement: FIG S2 [file msystems.00701-22-s0002.pdf]

**A**

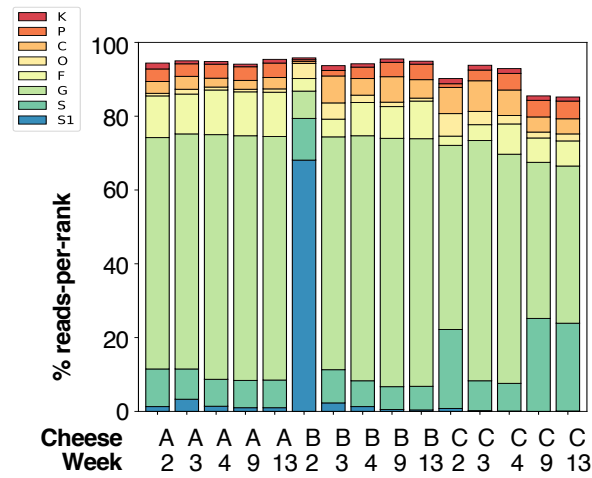

**B**

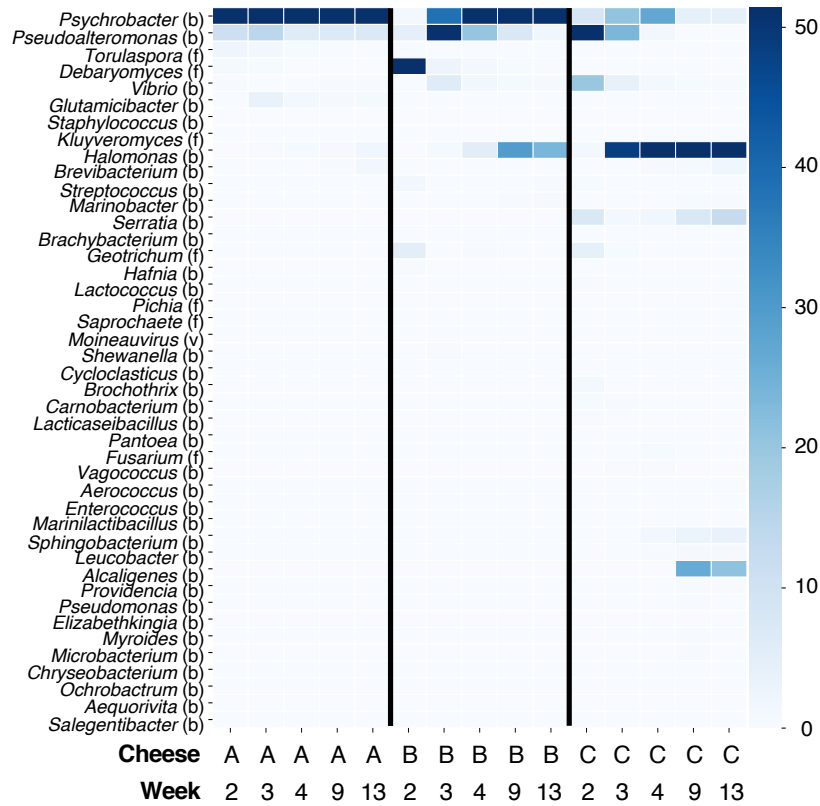

**C**

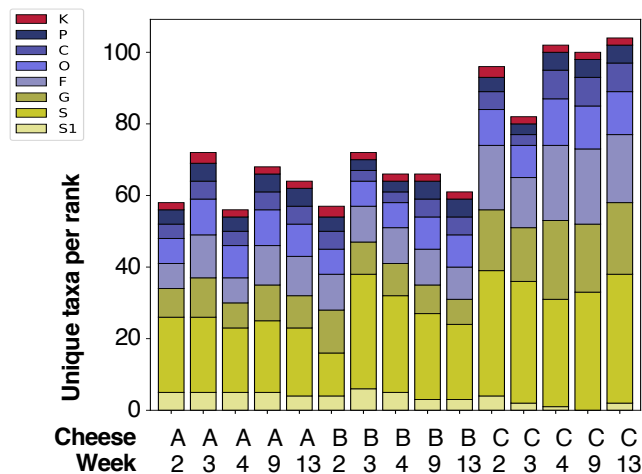

Supplement: FIG S3 [file msystems.00701-22-s0003.pdf]

A

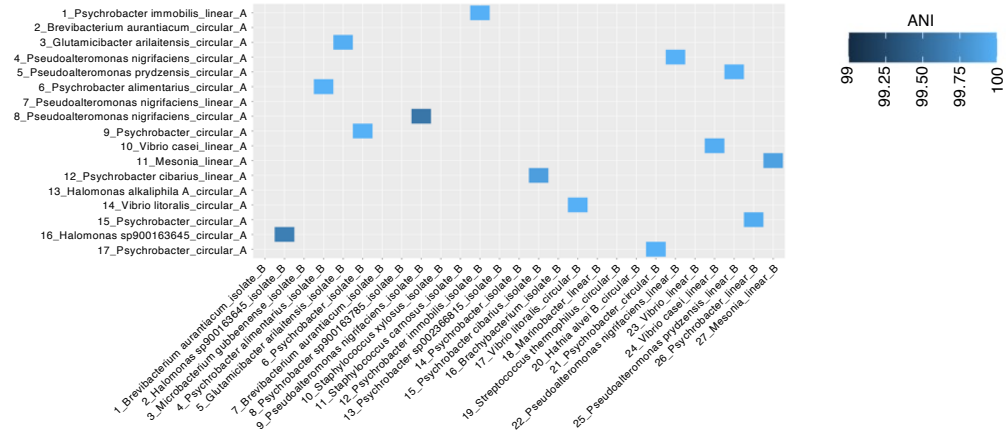

B

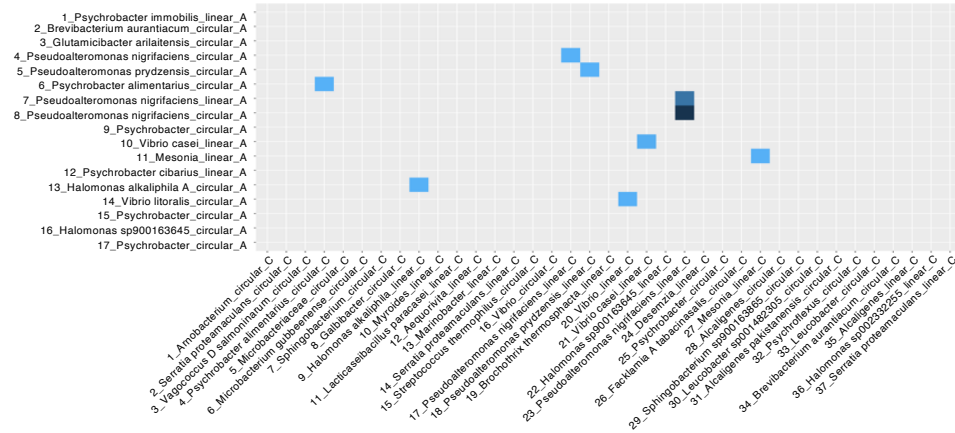

C

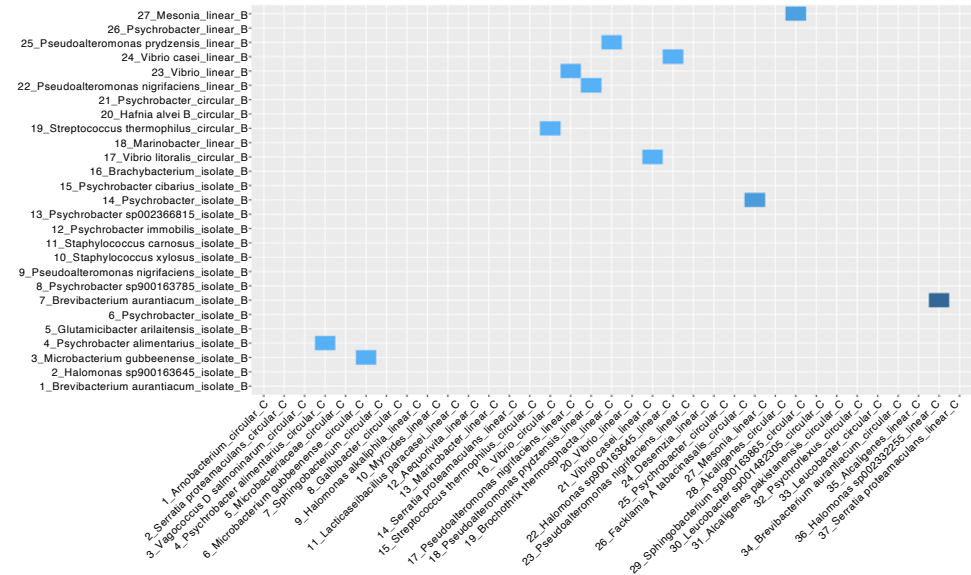

Supplement: FIG S4 [file msystems.00701-22-s0004.pdf]

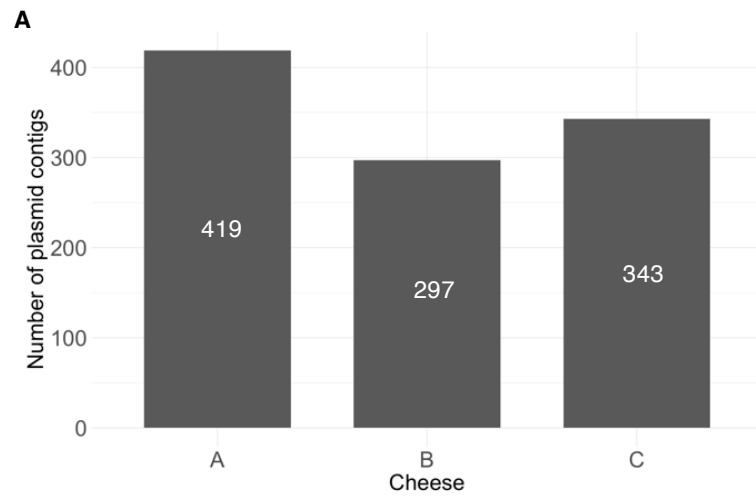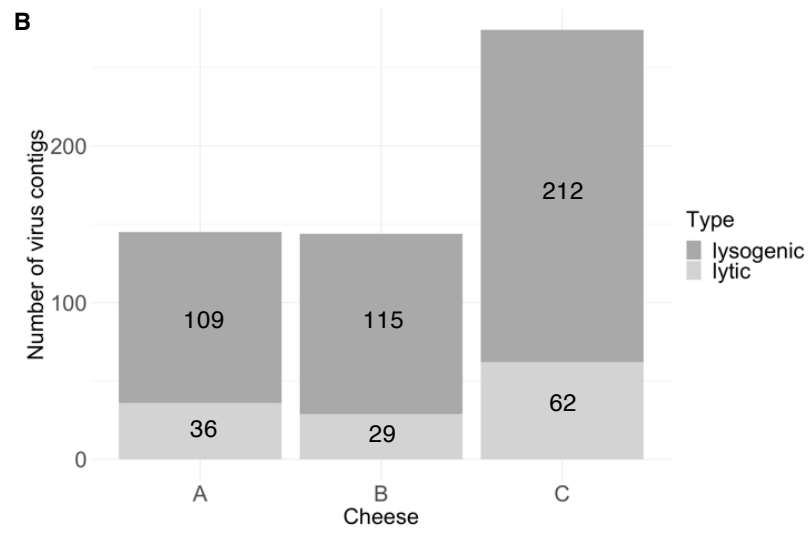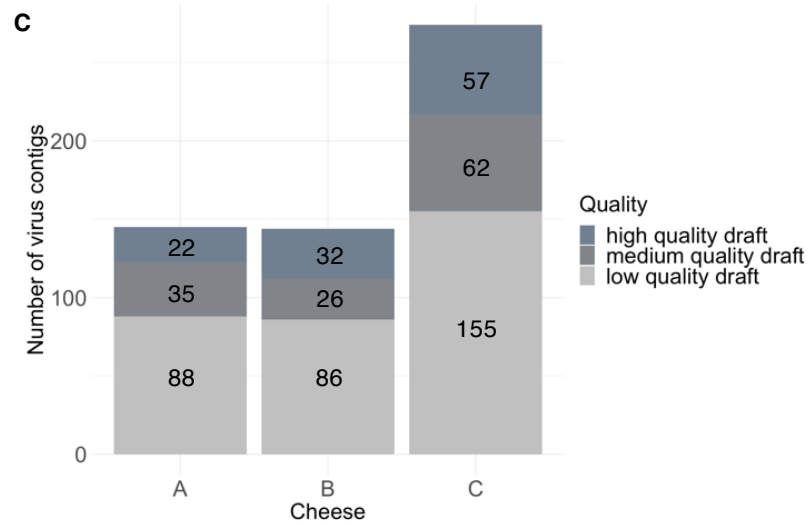

Supplement: FIG S5 [file msystems.00701-22-s0005.pdf]

**A**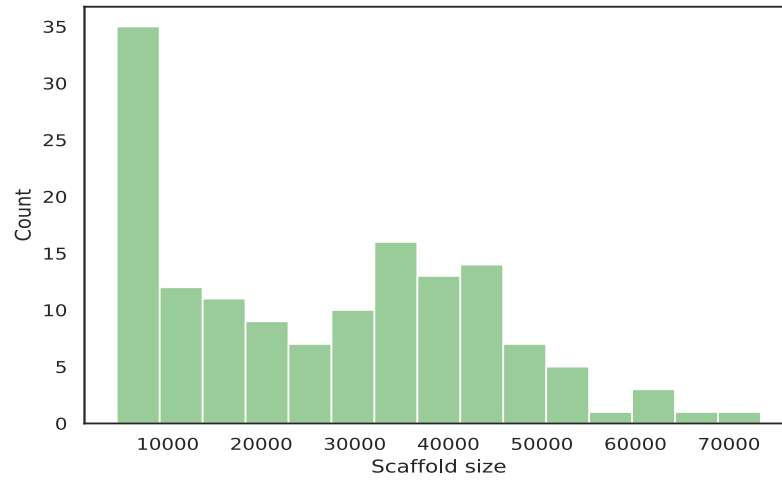**B**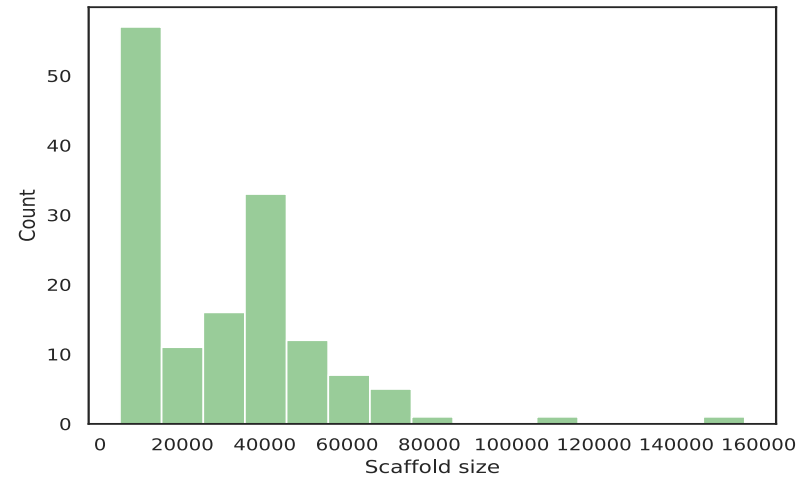**C**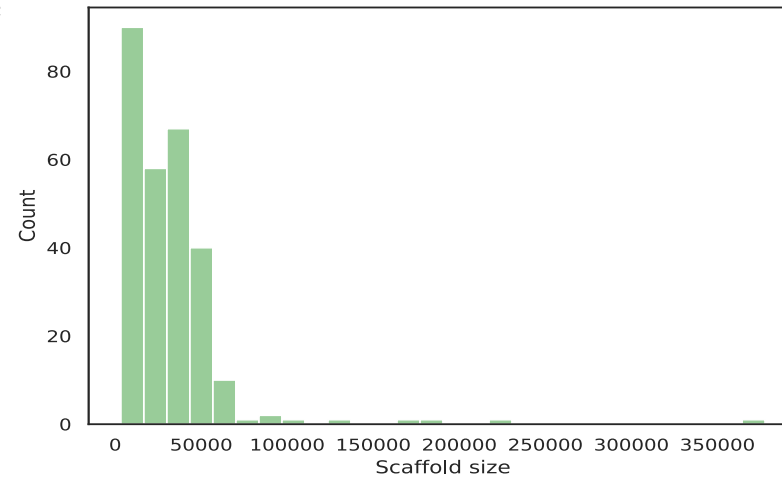

Supplement: FIG S6 [file msystems.00701-22-s0006.pdf]

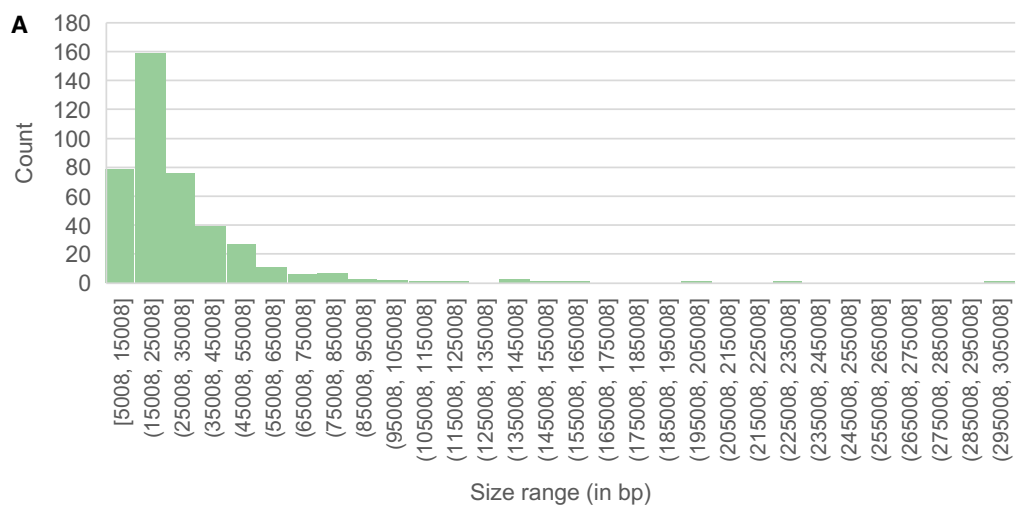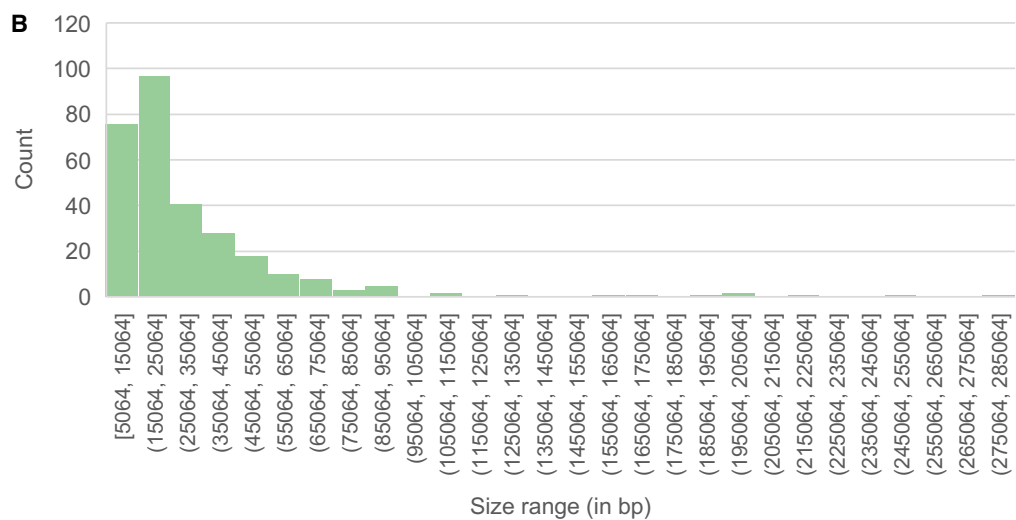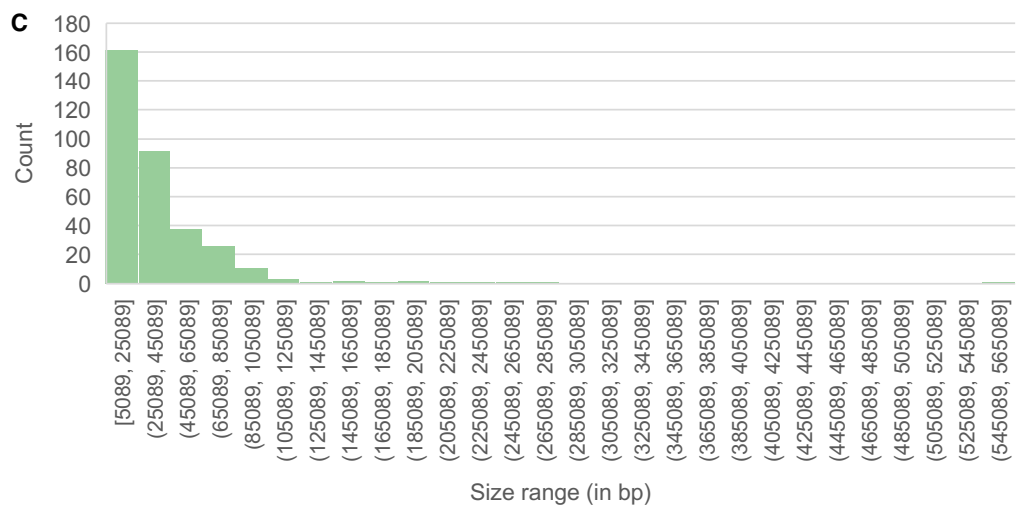

Supplement: FIG S7 [file msystems.00701-22-s0007.pdf]

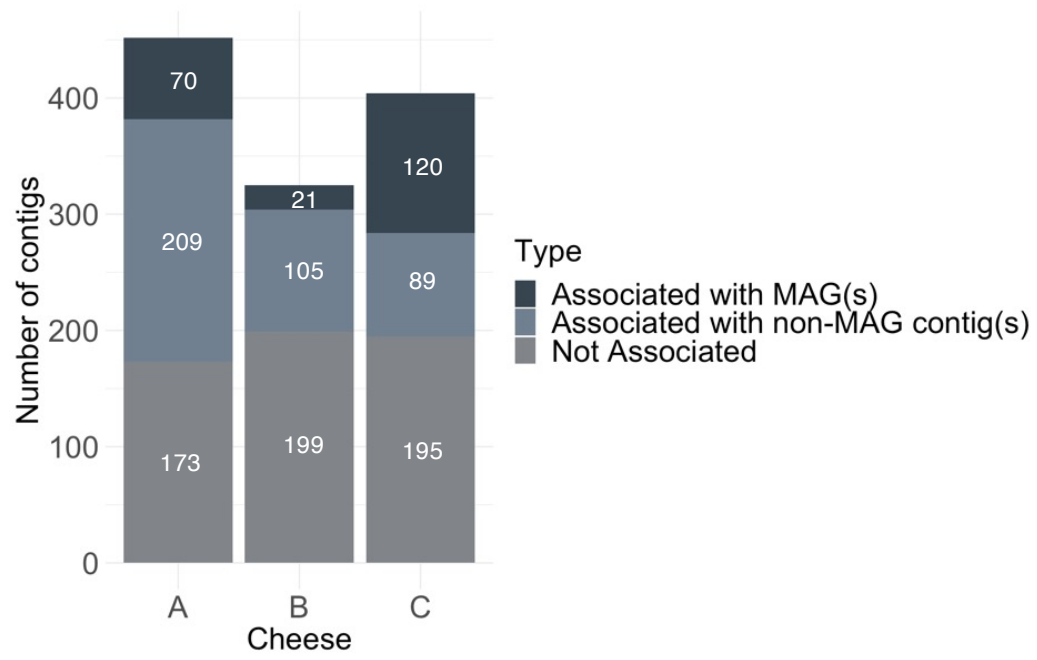

Supplement: FIG S8 [file msystems.00701-22-s0008.pdf]

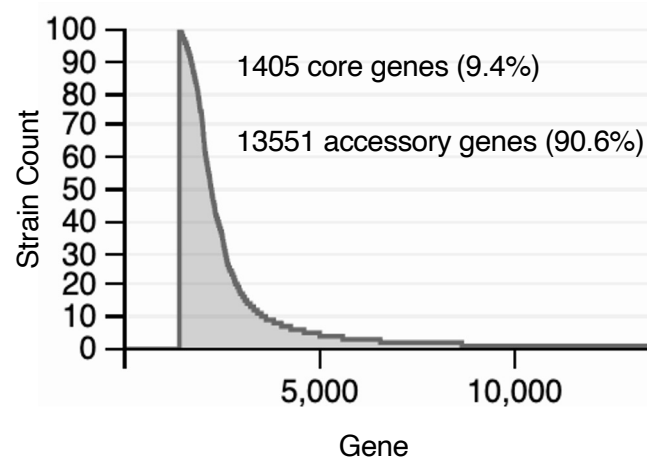

Supplement: FIG S9 [file msystems.00701-22-s0009.pdf]

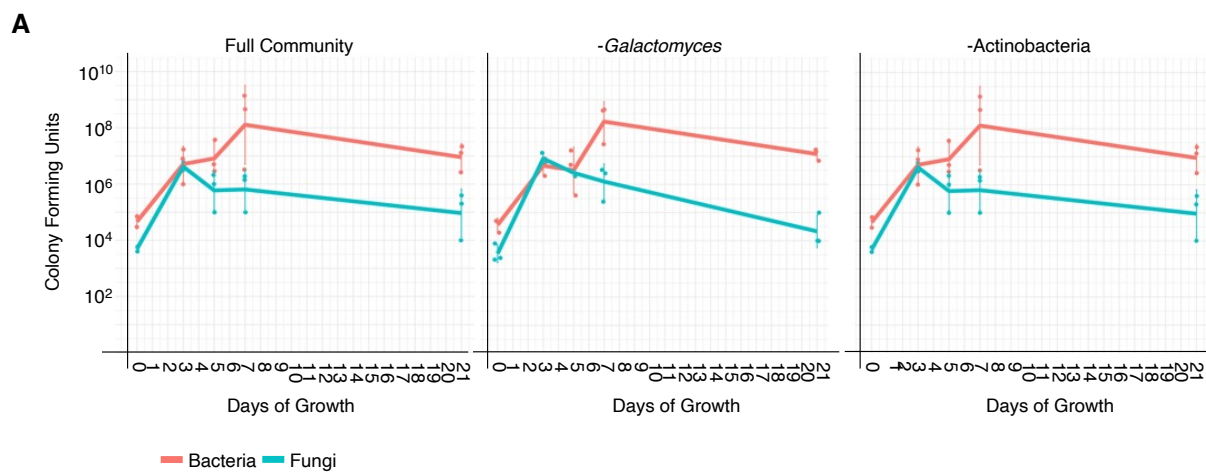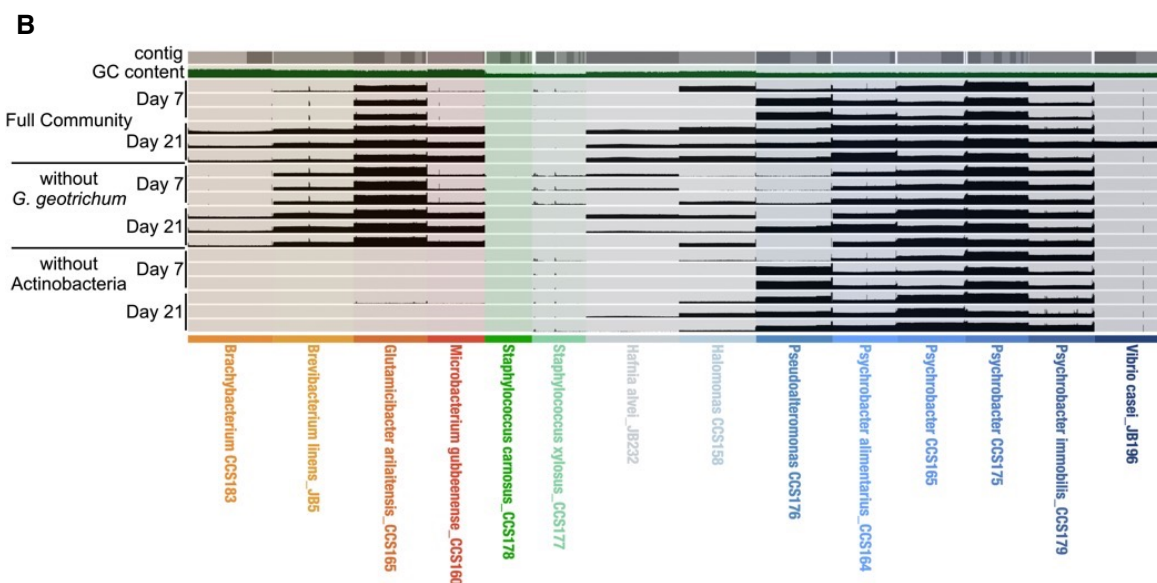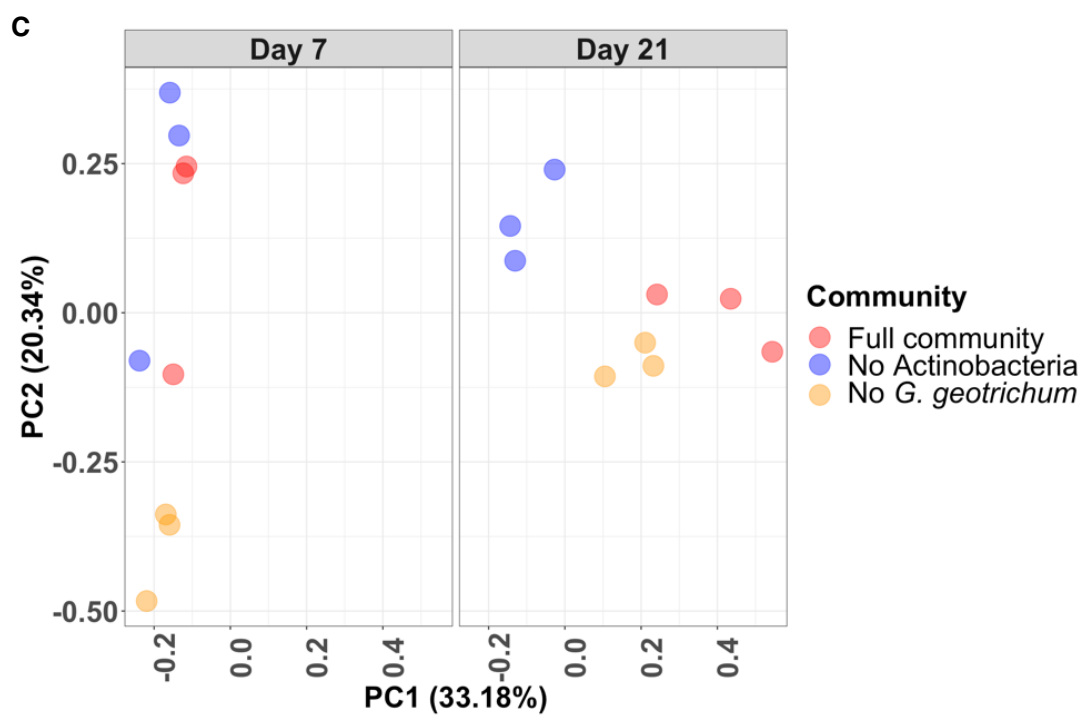

Supplement: FIG S10 [file msystems.00701-22-s0010.pdf]
